# Evaluation of the intestinal transmembrane transport and absorption characteristics of mangiferin in rats by the Ussing chamber technique

**DOI:** 10.1016/j.bbrep.2025.102334

**Published:** 2025-10-30

**Authors:** Yao-Yao Li, Chen-Huan Qiao, Shi-Dan Wang, Wan-Rong Dong, Wen-Bo Feng, Tong Liu, Yu-Xin Chen

**Affiliations:** Hubei Key Laboratory of Industry Microbiology, Cooperative Innovation Center of Industrial Fermentation (Ministry of Education & Hubei Province), School of Life and Health Sciences, Hubei University of Technology, Wuhan, 430068, China

**Keywords:** Mangiferin, Pharmacokinetics, Ussing chamber, Intestinal absorption characteristics

## Abstract

Mangiferin, one of the primary bioactive components in mango, exhibits diverse biological activities but relatively low absorption. Elucidating its absorption characteristics will facilitate the exploitation of its nutritional potential and the optimization of its bioavailability. This study employed liquid chromatography-mass spectrometry to determine mangiferin concentrations in rat plasma and used the Ussing chamber technique to evaluate intestinal absorption of mangiferin at varying concentrations, while comparing absorption differences across distinct intestinal segments. The results demonstrated that after oral administration of 200 mg/kg mangiferin to rats, the peak plasma concentration of 166.80 ng/mL was reached at approximately 2.1 h. The absorption of mangiferin in the ileum, cecum, and proximal colon of rats exhibited a concentration-dependent effect. Compared with the ileum, cecum, and mid-colon, mangiferin demonstrated significantly better absorption in the proximal colon (*p* < 0.05), indicating that the proximal colon serves as the primary intestinal site for mangiferin absorption into systemic circulation.

## Highlights

• The pharmacokinetic profile of mangiferin in rat plasma were obtained using LC-MS.
• Mangiferin's absorption along the intestinal axis was evaluated by Ussing chamber.
• The proximal colon was the primary site of mangiferin absorbing into the blood.

## Graphical abstract

**Figure:**
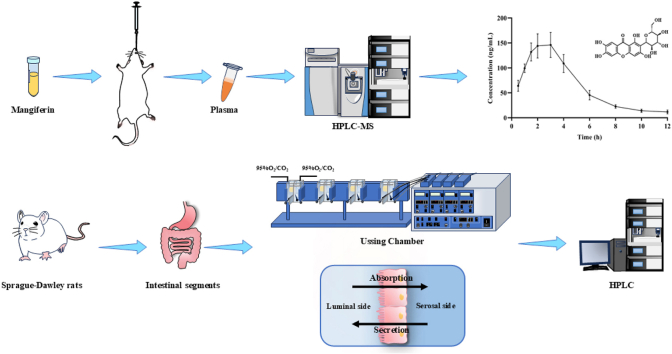

## 1 Introduction

Mangiferin is a natural compound that belongs to the Xanthone family. It is abundant in mangoes and is also widely present in plants of the Gentianaceae, Anacardiaceae, Iridaceae, and other families [1]. Mango (*Mangifera indica,* Linn) is regarded as one of the most popular fruits worldwide due to its excellent nutritional properties and delicious taste. Mangiferin, one of its primary active components, can reach a content of 0.253 mg/100 g in the fruit [2]. As a multifunctional natural compound, mangiferin possesses a unique polyhydroxy structure that endows it with remarkable efficacy in maintaining the body's redox balance [3]. Extensive research has demonstrated that mangiferin not only effectively prevents diabetes and its complications but also significantly protects the cardiovascular and nervous systems by regulating the synthesis of various biochemical substances in the body [4]. Meanwhile, plants rich in mangiferin can also be utilized as dietary supplements [5]. Novel bioactive compounds such as mangiferin can also play a significant role in the treatment of diseases. For instance, carminic acid has been shown to mitigate metabolic stress-induced non-alcoholic fatty liver disease [6]; Gingerols have been demonstrated to alleviate arthritis and pain, and certain gingerols have been implicated in the treatment of diabetes and various tumors [7], optimizing purification protocols to enable effective detection and drug development of these biomolecules remains a key research priority.

However, mangiferin is characterized by low solubility and lipophilicity, and first-pass effects from oral administration. These factors contribute to its suboptimal oral absorption and intestinal permeability, with a bioavailability as low as 1.2 % [8]. It has been reported that the C-glucoside in mangiferin can only be hydrolyzed in the intestine to generate aglycones with better bioavailability [9]. Therefore, studying its absorption and membrane permeability characteristics in the intestines is of great significance for promoting the development of mangiferin. Currently, research on the intestinal absorption of mangiferin primarily employs in situ perfusion models, Caco-2 cell models, and everted gut sac methods, but these approaches still face certain limitations [10]. However, these methods cannot investigate the absorption of mangiferin in specific intestinal segments or its absorption pattern along the intestinal tract.

The Ussing chamber was invented by the Danish scholar Hans Ussing when he was exploring the transport phenomenon of Na^+^ in epithelial cells. This model uses two half-chambers along with buffer solutions, temperature, and oxygen to construct a physiological environment close to that *in vivo*. After continuous improvements and enhancements, it is currently mainly used to study physiological characteristics such as ion transport, drug absorption, and the permeability of natural tissues and monolayer cells [11]. Due to its crucial role in the absorption of the body, the intestine has become one of the most frequently studied objects using the Ussing chamber technique.

The researchers used the oral administration results of *Salvia miltiorrhiza* Bunge and *Astragalus propinquus* Schischkin as references to compare the absorption parameters of the Caco-2 cell model, everted gut sac model, and Ussing chamber model. The results demonstrated that the Ussing chamber model was the closest to the actual *in vivo* absorption among the three models [12]. A study has utilized the Ussing chamber technique to compare the absorption effects of formononetin and ononin in different intestinal segments, demonstrating that the absorption of both substances in the large intestine segment is better than that in the small intestine and that formononetin has better absorption than ononin in the small intestine [13]. Another study showed that the Ussing chamber technique can be used to measure the intestinal transport and absorption of emodin. It was preliminarily determined that its transport is related to P-gp and MRP3 proteins [14].

In recent years, the use of dietary supplementation with natural bioactive compounds for health promotion has become a major public focus [15]. Mangiferin, a unique polyphenolic bioactive substance found in the widely popular and beloved *Mangifera indica* Linn., has attracted significant attention from researchers due to its notable physiological functions, such as metabolic regulation. Scientifically increasing dietary intake of mangiferin may emerge as a novel strategy for functional food development and nutritional intervention in chronic diseases. As a simpler and more accurate technique, the application of the Ussing chamber technique in research related to the intestinal absorption of mangiferin is relatively rare. Therefore, this study innovatively employs the Ussing chamber technique to accurately measure the absorption efficiency of mangiferin at varying gradient concentrations in the same intestinal segment. By comparing the absorption differences of mangiferin across different intestinal regions, it precisely identifies the optimal intestinal segment for mangiferin absorption. This provides crucial information regarding the absorption site of this nutrient, offering a reference for subsequent research on structural modification of mangiferin or its encapsulation to enhance bioavailability.

## 2 Materials and methods

### 2.1 Reagents

Mangiferin was purchased from Chengdu Alfa Biotechnology Co., Ltd. (Sichuan, China). Rutin was used as an internal standard (IS), purchased from Shanghai Yuanye Biotechnology Co., Ltd. (Shanghai, China), as well as sodium carboxymethylcellulose (CMC-Na) and ethylenediaminetetraacetic acid (EDTA). Chromatographic grade methanol and acetonitrile were purchased from Thermo Fisher Scientific (Shanghai, China). Other reagents were all of analytical grade and purchased from Sinopharm Chemical Reagent Co., Ltd. (Shanghai, China).

### 2.2 Pharmacokinetic studies

Five male Sprague-Dawley (SD) rats (230–260 g at 8 weeks of age) were provided by the Hubei Provincial Center for Disease Control and Prevention. They were housed under the conditions of temperature (20–25 °C), humidity (50–60 %), and a 12-h light/dark cycle. The animal experiments were conducted in the experimental animal center of Hubei University of Technology, in compliance with the guidelines of the Ethics Committee. All procedures involving animals were executed according to the Guide for the Care and Use of Laboratory Animals (NIH publication no. 85-23, eighth edition in 2011), and were authorized by the Animal Care and Use Committee of Hubei University of Technology (Wuhan, China) with approval number HBUTXM20250017. The rats were fasted for 12 h before the start of the experiment and had free access to water. Mangiferin was dissolved in 0.5 % sodium carboxymethylcellulose, and the rats were given a single intragastric administration (200 mg/kg). At 0.5, 1, 1.5, 2, 3, 4, 6, 8, 10, and 12 h after intragastric administration, the tail vein blood was collected into EDTA-containing tubes, centrifuged at 2500 rpm for 10 min, and the supernatant was taken and placed in a −20 °C refrigerator until analysis. At the end of the experiment, rats were euthanized by gradual displacement of air with CO_2_.

When detecting the sample concentration, rutin (IS) was added to the plasma, and the plasma was extracted three times with acetonitrile: acetic acid (9:1). The supernatants were centrifuged and combined to obtain plasma samples at each time point. A standard curve was prepared using blank plasma with added gradient concentrations of mangiferin and IS. Each sample was dried with nitrogen on a heating plate at 40 °C, and resuspended in methanol: water (1:1).

Quantitative analysis was carried out using a Thermo Fisher Q-Exactive high-resolution liquid chromatography-mass spectrometry (LC-MS) instrument. The chromatographic separation was performed on an Agilent ZORBAX SB-C_18_ column (2.1 × 50 mm) maintained at 25 °C, using mobile phase A (0.1 % formic acid in water) and mobile phase B (0.1 % formic acid in methanol) at a flow rate of 0.2 mL/min. The gradient elution program was set as follows: 0–1 min, 10 % B; 1–5 min, linear gradient from 10 % to 90 % B; 5–8 min, 90 % B; 8–8.1 min, rapid return to 10 % B; and 8.1–10 min, column re-equilibration at initial conditions (10 % B). Mass spectrometry conditions: The ion transfer tube temperature was set at 350 °C, vaporizer temperature at 300 °C, with sheath gas pressure of 35 arb and auxiliary gas pressure of 10 arb. A spray voltage of 3.5 kV was applied for negative ion mode detection. Targeted-SIM-ddMS2 mode was employed for the acquisition of mangiferin and rutin, with a collision energy of HCD 15.

### 2.3 Experimental methods for the Ussing chamber

The intestinal segments of male Sprague-Dawley (SD) rats were taken and washed in Krebs-Ringer buffer (pH = 7.4), which was composed of the following substances: 117 mM NaCl, 24.8 mM NaHCO_3_, 4.7 mM KCl, 1.2 mM MgCl_2_, 1.2 mM KH_2_PO_4_, 2.56 mM CaCl_2_·2H_2_O, and 11.1 mM C_6_H_12_O_6_. Tissue fragments of the ileum, cecum, proximal colon, and mid-colon were obtained according to previous procedures [16]. Tissue clips were used to vertically fix the segments between the two chambers in the Ussing Chamber system. Circulating water at 37 °C and a mixed gas (95 % O_2_, 5 % CO_2_) were introduced to simulate the physiological environment required by the intestinal segments. A pair of electrodes made of KCl agar bridges was used to measure the voltage between the two chambers, and the current was monitored by a voltage clamp amplifier (Physiological Instruments VCC MC6). The transepithelial electrical resistance (TEER) was calculated. The data in which the decrease in TEER of the tissues at the end of the experiment was less than 15 % of the initial TEER were considered to be useable [17].

### 2.4 Absorption and secretion experiments of mangiferin

After euthanized, rats were dissected to harvest intestinal segments, including the ileum, cecum, and proximal colon. The intestinal segments were opened along the mesenteric border and thoroughly rinsed. Under a stereomicroscope, the outer serosal and muscle layers were carefully removed to obtain a standard mucosa-submucosa specimen. The preparations were then mounted in tissue clamps with a permeation area of 0.5 cm^2^ and loaded into the Ussing chamber system. 3 mL of buffer solution was added to both sides simultaneously, and incubated for 10 min. The preheated mangiferin solutions with gradient concentrations (prepared with buffer solution) and Krebs-Ringer buffer were used to replace the solutions simultaneously after the above incubation. Samples (600 μL) were collected from the serosal side at 30, 60, 90, and 120 min after the experiment began. The concentration of mangiferin at each time point was determined by HPLC analysis. Meanwhile, the pre-warmed buffer solution was replenished to the serosal side to maintain the original volume.

The Thermo Fisher U3000 high-performance liquid chromatography system (Thermo Fisher Scientific, Shanghai, China) and C_18_ column (COSMOSIL, 4.6 mm × 250 mm, 5 μm) were used for chromatographic separation. The mobile phase consists of 0.1 % formic acid aqueous solution (A) - acetonitrile (B) (88:12, v/v), with isocratic elution for 15 min. The column temperature is maintained at 30 °C, the flow rate is 1.0 mL/min, and the detection wavelength is set at 254 nm.

The relevant absorption parameters, such as the Cumulative absorption amount Q, Absorption rate constant Ka, and Apparent permeability coefficient Papp, were calculated with reference to the previous studies [18]. Additionally, mangiferin at a concentration of 600 μg/mL was set up to compare the absorption effects of mangiferin in the ileum, cecum, proximal colon, and mid-colon of rats. In the mangiferin secretion experiment, mangiferin was absorbed by the intestine from the serosal side to the luminal side, and samples were taken from the luminal side to measure its concentration, thereby calculating the secretion amount of mangiferin in different intestinal segments of rats.

## 3 Data analysis

The pharmacokinetic parameters were calculated using the Drug and Statistics software (version 2.0) of the Chinese Society of Mathematical Pharmacology. IBM SPSS Statistics 25.0 was employed for data analysis, and all the results were expressed as the mean ± standard error of the mean (SEM). One-way ANOVA with the least significant difference test was used between multiple groups, and *p* < 0.05 was considered a statistically significant difference.

## 4 Results and discussion

### 4.1 Pharmacokinetic parameters of mangiferin

The concentration of mangiferin in rat plasma was determined by LC-MS. The obtained standard curve was y = 0.168x + 0.2104, and it showed a good linear relationship within the tested concentration range of 0.5–100 ng/mL (R^2^ > 0.999). In our experiments, the retention times of mangiferin and the internal standard were well-separated. Mangiferin demonstrated satisfactory analytical performance across low, medium, and high concentrations (1, 20, and 80 ng/mL). The relative standard deviations (RSD) for precision were all below 15 %, while the relative errors (RE) for accuracy were all below 18 %. In a pharmacokinetic study employing LC-MS/MS to determine D-4-cystine in mice, it was considered acceptable to set the precision (RSD, %) and accuracy (RE, %) limits at 20 %, and the results of this study were consistent with these criteria [19]. The method showed consistent recovery rates of 98.2 ± 10.2 %, 100.6 ± 12.5 % and 96.2 ± 4.1 % at these three concentration levels, with all RSD values maintained under 13 %. The relative matrix effect evaluation showed that the coefficient of variation for mangiferin at the three concentrations was less than 10 %. The accuracy of mangiferin under room temperature, 4 °C storage, and freeze-thaw cycle conditions ranged from 93.3 % to 109.8 %. Overall, the accuracy of the method is favorable.

The mangiferin plasma concentration in rats following oral gavage of mangiferin is shown in Table 1, and the pharmacokinetic parameters are presented in Table 2. After oral administration at a dose of 200 mg/kg, mangiferin was rapidly absorbed and reached its peak at around 2.1 h, then was quickly eliminated and reached its half-life at around 2.6 h. In addition, the peak concentration of mangiferin was 166.80 ng/mL. A study on the absorption kinetics of mangiferin in humans demonstrated that the pharmacokinetics of mangiferin were nonlinear; the absorption increased with the increase in the dose [20]. The T_max_ of mangiferin absorption observed in rats in this study was essentially consistent with the values obtained at the oral administered doses of 0.1 g and 0.3 g in the investigation.

**Table 1:** Mangiferin plasma concentrations in rats following oral gavage of 200 mg/kg mangiferin (n = 5).

Time (h)	Concentration (ng/mL)
0.5	64.06 ± 11.00
1	99.35 ± 8.61
1.5	131.95 ± 18.00
2	144.06 ± 24.12
3	146.14 ± 24.97
4	108.95 ± 18.04
6	45.37 ± 9.57
8	22.65 ± 3.56
10	14.07 ± 3.15
12	8.19 ± 3.08

**Table 2:** Pharmacokinetic parameters of rats after mangiferin administration.

PK parameters	i.g.(200 mg/kg)	Unit
T_max_	2.10 ± 0.41	h
t_1/2_	2.60 ± 0.45	h
C_max_	166.80 ± 19.64	ng**·**mL^−1^
AUC_0-t_	728.13 ± 74.06	h**·**ng**·**mL^−1^
AUC_0-∞_	773.20 ± 72.38	h**·**ng**·**mL^−1^
CLz/F	270.52 ± 25.50	L**·**h^−1^**·**kg^−1^
Vz/F	1031.56 ± 214.68	L**·**kg^−1^
MRT_0-t_	3.73 ± 0.15	h

### 4.2 Absorption of mangiferin with gradient concentrations in intestinal segments

**Fig. 1:**
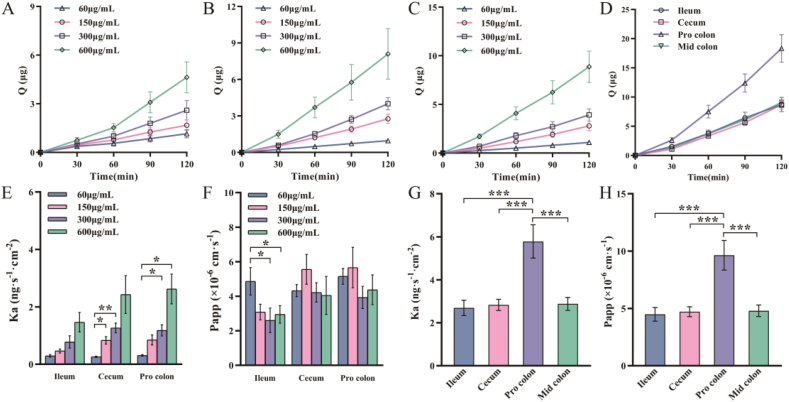
Cumulative absorption amounts (Q) of mangiferin at four concentrations in three intestinal segments: (A) Ileum; (B) Cecum; (C) Proximal colon. (D) Cumulative absorption amounts of 600 μg/mL mangiferin in four intestinal segments; (E) Absorption rate constant (Ka) and (F) apparent permeability coefficient (Papp) of mangiferin at four concentrations in three intestinal segments; (G) Absorption rate constant and (H) apparent permeability coefficient of 600 μg/mL mangiferin in four intestinal segments. The data are expressed as mean ± SEM (n = 6). ∗*p* < 0.05,∗∗*p* < 0.01, ∗∗∗*p* < 0.001.

As shown in Fig. 1A–C, the concentration of mangiferin was directly proportional to the cumulative absorption amount in the intestinal (ileum, cecum, proximal colon) segments. The absorption amount of 600 μg/mL mangiferin in the three intestinal sites was higher than that of other concentrations at each time point. Moreover, as the concentration increased, the absorption rate constant Ka also gradually increased in Fig. 1E, which proved that the absorption effect of mangiferin in the intestine was directly proportional to the concentration.

According to the existing research, Papp <1 × 10^−6^ cm s^−1^ indicates poor absorption, 1 × 10^−6^ cm s^−1^ < Papp <1 × 10^−5^ cm s^−1^ indicates moderate absorption, and Papp >1 × 10^−5^ cm s^−1^ is considered good absorption [21]. Fig. 1F shows the apparent permeability coefficient (Papp) of mangiferin at different concentrations. All the Papp values were greater than 1 × 10^−6^ cm s^−1^, indicating that the absorption of mangiferin in the intestine was moderate. Among the three intestinal segments, the intestinal Papp of mangiferin at low concentrations (60, 150 μg/mL) was generally higher than that at high concentrations (300, 600 μg/mL). However, in the ileum, the Papp of 60 μg/mL mangiferin was higher than that of the other three concentrations. In contrast, in the cecum and proximal colon, the permeability coefficient of 150 μg/mL mangiferin was relatively higher. The absorption of flavonoid compounds is known to depend on factors such as their physicochemical properties, intestinal transporter regulation, gut physiological status, and metabolic enzyme activity [22]. The data demonstrate a concentration-dependent, non-linear increase in the absorption rate constant (Ka) of mangiferin at elevated concentrations, concomitant with a reduction in its apparent permeability coefficient (Papp). This suggests that the absorption of mangiferin may be associated with metabolic enzyme saturation and transporter activity. Mangiferin exhibits poor intestinal barrier permeability due to its strong hydrophobicity and low aqueous solubility. Future studies may employ Ussing chamber technology to enhance its intestinal absorption through either structural modification or transporter regulation strategies.

### 4.3 Absorption of mangiferin in different intestinal segments

In order to further analyze the absorption characteristics of mangiferin along the intestinal tract, the present study compared the absorption of mangiferin in different intestinal segments. As shown in Fig. 1D, within 2 h of the experiment, the cumulative absorption amounts in all four intestinal segments increased with the increase in time, among which the proximal colon had the largest increase. According to Fig. 1G and H, among the four intestinal segments, the proximal colon had the fastest absorption rate and the best absorption effect (*p* < 0.001). It was found through the in situ perfusion model that both the effective permeability and the drug absorption rate limitation of mangiferin in the colon were greater than those in the ileum segment, which was consistent with the results of our study [23]. However, in another study on the intestinal absorption of mangiferin using the everted gut sac method, both of these two parameters of the ileum segment were greater than those of the colon segment [24]. It is speculated that this difference is caused by the different intestinal physiological states in different experimental methods.

**Fig. 2:**
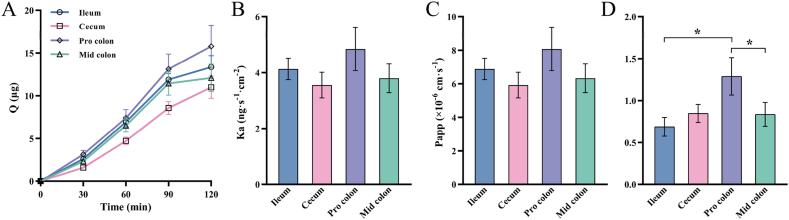
Secretion parameters of 600 μg/mL mangiferin in four intestinal segments: (A) Cumulative secretion amount (Q); (B) Secretion rate constant (Ka); (C) Apparent permeability coefficient (Papp); (D) Ratio of Absorption to Secretion. The data are expressed as mean ± SEM (n = 6). ∗*p* < 0.05.

In order to dynamically analyze the absorption status of mangiferin along the intestine, this study conducted a secretion study with 600 μg/mL mangiferin and calculated the absolute absorption in the intestinal segments. The results are shown in Fig. 2A–C. The increased amplitude of the cumulative secretion amount of mangiferin in the four intestinal segments had no obvious difference, and there were also no significant differences in the secretion rate constant and the apparent permeability coefficient. By dividing the absorption parameters by the secretion parameters, the result shown in Fig. 2D was obtained. It can be seen that the absolute absorption value of the proximal colon was greater than that of the other three segments, and there were significant differences between the proximal colon and the ileum, as well as the proximal colon and the middle colon (*p* < 0.05).

**Fig. 3:**
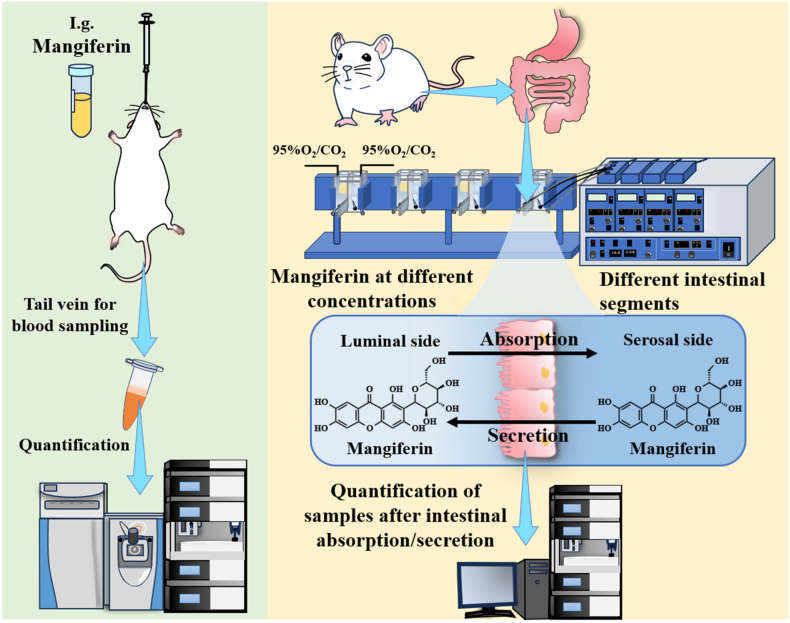
Schematic diagram of mangiferin pharmacokinetics and intestinal absorption.

Fig. 3 illustrates the schematic diagram of the research process. In summary, SD rats were selected as experimental subjects, and LC-MS technology was utilized to conduct *in vivo* pharmacokinetic experiments of mangiferin. Additionally, the Ussing chamber was combined with HPLC to assess the absolute absorption of mangiferin in various intestinal segments.

**Fig. 4:**
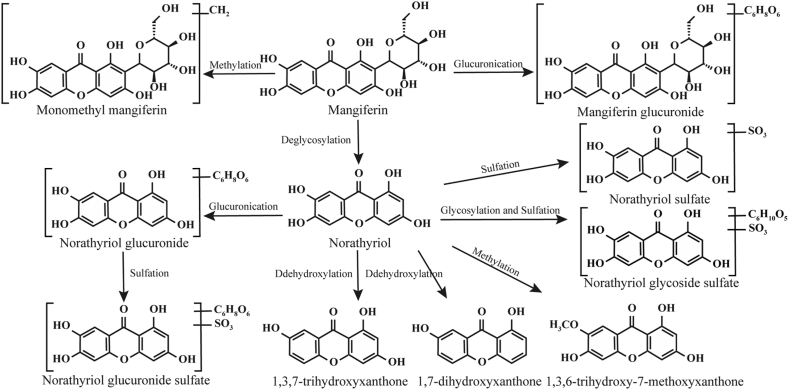
The potential metabolic pathways of mangiferin as summarized from the references.

Fig. 4 summarizes the reported metabolic pathways of mangiferin [9,25,26]. Based on this metabolic map, the future work of this study will include the following: after transporting mangiferin for a period of time using the Ussing chamber, the solutions from the luminal and serosal sides were collected for mass spectrometry analysis. The compositional differences between the solutions from both sides were compared to identify the specific metabolites of mangiferin resulting from its transport across the intestinal segment. This investigation will contribute to elucidating mangiferin's transformation after its transport across the intestinal membrane, lay a theoretical foundation for its development and application as a functional dietary ingredient.

## 5 Conclusions

This study has clarified the pharmacokinetic characteristics of mangiferin in rat plasma after oral administration. It was demonstrated by the Ussing chamber technique that the absolute absorption of mangiferin in the intestine is affected by its concentration and that the absolute absorption effect varies in different intestinal segments, among which the absolute absorption effect in the proximal colon is relatively the best. This provides important metabolic kinetic evidence for further research on the development of functional foods containing mangiferin and its health applications. At the same time, it also verifies the applicability of the Ussing chamber technique in studying the intestinal absorption characteristics of mangiferin, offering new research insights for optimizing food formulations and consumption methods to enhance its bioavailability and nutritional efficacy.

## CRediT authorship contribution statement

**Yao-Yao Li:** Data curation, Formal analysis, Investigation, Software, Validation, Writing – original draft. **Chen-Huan Qiao:** Data curation, Investigation, Software. **Shi-Dan Wang:** Data curation, Investigation, Software. **Wan-Rong Dong:** Data curation, Investigation. **Wen-Bo Feng:** Investigation. **Tong Liu:** Investigation. **Yu-Xin Chen:** Conceptualization, Funding acquisition, Methodology, Project administration, Resources, Supervision, Writing – review & editing.

## Declaration of competing interest

The authors declare that they have no known competing financial interests or personal relationships that could have appeared to influence the work reported in this paper.

## Data Availability

Data will be made available on request.
